# Digital Light Processing (DLP) 3D Printing Fabrication of Hydrophobic Meshes Incorporating Fluorinated and Silicone-Based Acrylates Combined with Surface Engineering: Comparison of Their Oil–Water Separation Efficiency

**DOI:** 10.1021/acsomega.4c07193

**Published:** 2024-11-29

**Authors:** Wai Hin Lee, David Haddleton

**Affiliations:** Department of Chemistry, University of Warwick, Coventry CV4 7AL, U.K.

## Abstract

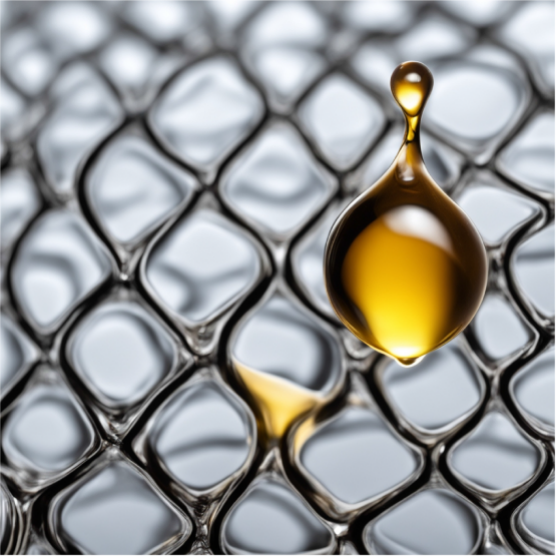

Hydrophobic materials have been fabricated by DLP vat photopolymerization of isobornyl acrylate-based resins with chemical modification and/or surface geometry engineering. Fluorinated and polydimethylsiloxane (PDMS)-based acrylic monomers are used for chemical modification and are incorporated into the printed materials. The water wettability was significantly reduced and plateaued with as low as 5% (w/w) of the auxillary hydrophobic monomer. Regarding surface geometry, meshes with different pore sizes are 3D printed, and the surface hydrophobicity increased with the pore size. We compare the oil–water separation efficiency of the 3D-printed meshes hydrophobized by these three approaches. It was found that the isobornyl acrylate-based resin already demonstrated separation at the optimum pore size. Modification with PDMS showed a further improvement in separation efficiency, whereas no significant increase was observed by use of the fluorinated monomer. This highlights that careful design of surface geometry should be considered to avoid the use of environmentally unfriendly and potentially toxic chemicals when making hydrophobic materials.

## Introduction

Incorporation of hydrophobicity into materials is useful for many applications, including increasing water repellency, moisture barriers, self-cleaning, anticorrosion, etc.^1−5^ Polyalkylsiloxane and fluorine-containing monomers are two common ways to introduce hydrophobicity into materials, and indeed these have been used to give attractive properties to consumers.^6−8^ It is noted that the use of both F and Si into materials is notoriously harmful to the environment due to the low biodegradability of the fluorinated carbons/PDMS moieties, and F-containing materials are often termed “*forever chemicals*” due to undesirable properties.^9,10^ Moreover, fluorinated hydrocarbons can degrade to produce carcinogenic fluorotelomers and/or perfluorinated acids, which can cause further detrimental health and environmental effects.^11,12^ Alternatively, hydrophobic materials can be obtained by surface engineering to introduce porosity, roughness, or patterning. This sophisticated design approach has long been demonstrated in nature, for example, with lotus leaves and the water strider.^13−15^

With the emergence of 3D printing techniques, hydrophobic objects with refined structures can be fabricated easily.^16−26^ Thickett et al. used stereolithography to construct 3D objects via radical thiol–ene chemistry with postmodification of the excess thiol group at the surface via surface-grafted RAFT polymerization of 2,2,2-trifluoroethyl methacrylate to enhance hydrophobicity.^27^ Helmer et al. used perfluoropolyether (PFPE)-methacrylate in conjunction with perfluorooctanol as a porogen to induce phase separation upon photopolymerization and form a nanoscale porous structure to obtain superhydrophobic surfaces.^18^ Levkin demonstrated a similar strategy with commercially available butyl acrylate and decanol as monomer and porogen, and attained similar superhydrophobicity and droplet pinning effects.^22,28^ Ameduri reported the use of diacrylates based on vinylidene fluoride (VDF) and perfluoromethyl vinyl ether (PMVE) and utilized for photo-cross-linking into fluorinated networks.^29,30^ De Simone used fluorinated-functionalized perfluoropolyethers that were photocured into hydrophobic networks for the production of microfluidic devices as nonswelling replacements for PDMS and as hard abrasive cross-linked materials to reduce fouling.^31,32^ Bongiovanni has also reported the UV curing of perfluoroalkyl and perfluoropolyether fluorinated difunctional and monofunctional (meth)acrylic monomers and their effect on film properties.^33,34^

3D vat printing offers advantages in surface engineering with the construction of well-defined geometry on the surface to enhance hydrophobicity via tailoring the surface porosity and/or pattern. Vat photopolymerization, although being inexpensive and with low technical requirements, has been relatively less explored in this aspect due to the poorer resolution (ca. 0.05 mm) compared to other printing techniques.^35,36^ Kaur et al. used hydrophobically modified silica nanoparticles as a filler, with the combination of micropillars on the surface, to fabricate hydrophobic objects without the necessity of conventional hydrophobic monomers.^24^ Danielak et al. 3D-printed sharp tips with hydrophilic PEGDA (Mwt: 575 g mol^–1^) and showed a large increase of the water contact angle from 65 ^°^ to 112.5°.^37^ Despite relatively less attention being paid, these works demonstrate the potential of vat photopolymerization to fabricate hydrophobic objects via surface engineering without the use of hazardous materials.

In this present study, we have used DLP 3D printing to construct porous structures composed of isobornyl acrylate with and without the addition of both PDMS and fluorine-containing monomers. The water wettability of the 3D-printed objects was evaluated to compare the hydrophobicity introduced by chemical modification and surface geometry engineering. We also examined the necessity of hydrophobic monomers in 3D-printed meshes for oil–water separation. This highlights the importance of careful design of the surface geometry in addition to the chemical composition in hydrophobic materials.

## Experimental Details

### Materials

Isobornyl acrylate (IBoA), hexanediol diacrylate (HDDA), alizarin, and tributyrin were purchased from Sigma-Aldrich. Tridecafluorooctyl acrylate (TDFA) and tridecafluorooctyl methacrylate (TDFMA) were obtained from Chemours. 2,2,2-Trifluoroethyl acrylate (TFEA) and 2,2,2-trifluoroethyl methacrylate (TFEMA) were purchased from Sigma-Aldrich. Phenylbis(2,4,6-trimethylbenzoyl)phosphine oxide (BAPO) was supplied by the Tokyo Chemical Industry. Monomethacrylate-terminated polydimethylsiloxane was supplied (PDMS-A, Mwt: 680 g mol^–1^) by Unilever.

### Formulations

The resin formulations used are summarized in Tables 1 (fluorinated monomers) and 2 (PDMS-MA). In brief, all the components were mixed prior to being stirred vigorously, followed by sonication until transparent liquid formulations were obtained. All resins were stored in amber jars under ambient conditions until use.

**Table 1:** Formulations of the Isobornyl Acrylate-Based Resins with Fluorinated Monomers

	IBoA/g	F-monomer/g	HDDA/g	Alizarin/mg	BAPO/g
1	16		4	6	0.2
2	15.9	TDFA 0.1			
3	15.8	TDFA 0.2			
4	15.6	TDFA 0.4			
5	15.4	TDFA 0.6			
6	15	TDFA 1			
7	12	TDFA 4			
8	3.4	TDFA 12.6			
9	15	TDFMA 1			
10	15	TFEA 1			
11	15	TFEMA 1			

**Table 2:** Formulations of the Isobornyl Acrylate-Based Resins with PDMS-MA

	IBoA/g	PDMS-A/g	HDDA/g	Alizarin/mg	BAPO/g
12	15.9	0.2	4	6	0.2
13	15.8	0.4			
14	15.6	0.6			
15	15.4	0.8			
16	15	1			
17	14	2			

### 3D Printing

All printing was performed by using an inexpensive “*Anycubic D2 DLP*” printer. In brief, a 30 × 30 × 3 mm^3^ board and 10 × 10 × 5 mm^3^ meshes with various pore diameters (0.5, 0.6, 0.75, 1, 1.5 mm) and a separation distance of 0.5 mm longer than the diameter were designed using “*Onshape CAD software*”. The .stl file was sliced using Anycubic Photon Workshop software with a layer thickness of 50 mm and exposure times of 10 and 60 s for normal layer and bottom layer, respectively, for acrylate, and 30 and 60 s for methacrylate. All printed objects were subsequently washed with isopropanol and postcured under UV (λ = 405 nm) for 20 min. A typical .stl file and image of the printed mesh are shown in Figure S1

### Contact Angle Measurements

Contact angles were measured using a Krüss drop shape analysis system DSA100. In a typical experiment, a 1 mL Braun Inkjet syringe was equipped with a FISNAR 23G blunt-end tip needle. Water (20 μL) was dropped onto the surface of the 3D-printed board. Subsequently, the needle was lowered to the core of the droplet, and more water was added at a constant rate of 0.5 μL s^–1^. Images were taken for 30 s at 5 fps. The contact angles were automatically calculated from Young–Laplace fitting with a manual baseline. The advancing and receding angles were recorded at the last frame before and the first frame after the lateral length of the droplet changed as more water was added. The surface tension is calculated from eqs 1 and 2 by the software. Typical images of each sample are shown in Figures S2–S7
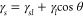
1
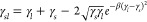
2where γ_l_, γ_s_, and γ_s1_ are the surface tension of water (72.8 mN m^–1^ at 25 °C), solid substrate, and the interfacial tension, respectively; β = 0.0001247° is an empirical correcting constant suggested by the instrument.

### Scanning Electron Microscopy (SEM) and Energy-Dispersive X-Ray (EDX) Analysis

SEM and EDX were performed on a Zeiss Gemini field emission scanning electron microscope with associated energy-dispersive X-ray spectroscopy. No coating was applied to preserve the chemical composition and facilitate the EDX elemental analysis. An In-lens secondary electron detector and a Type II secondary electrons detector were used for surface characterization and EDX elemental analysis, respectively.

### Fourier Transform Infrared Spectroscopy (FTIR)

FTIR-ATR spectroscopic data were acquired on a Bruker ALPHA II. The acquisition range was 400–4000 cm^–1^ with a resolution of 4 cm^–1^. Sixteen scans were accumulated for both baseline and sample measurements.

### Oil–Water Separation by 3D-Printed Objects

The oil–water separation was performed by pouring a mixture of tributyrin (3 mL) and water (3 mL) dyed with alizarin for visual aid (pink and yellow in the aqueous and oil phaseS, respectively). The separation efficiency was evaluated by the mass of the oil and water passing through the mesh without additional pressure.

## Results and Discussion

### Enhancing Hydrophobicity by Incorporation of Fluorinated and Silicone Monomers

**Figure 1:**
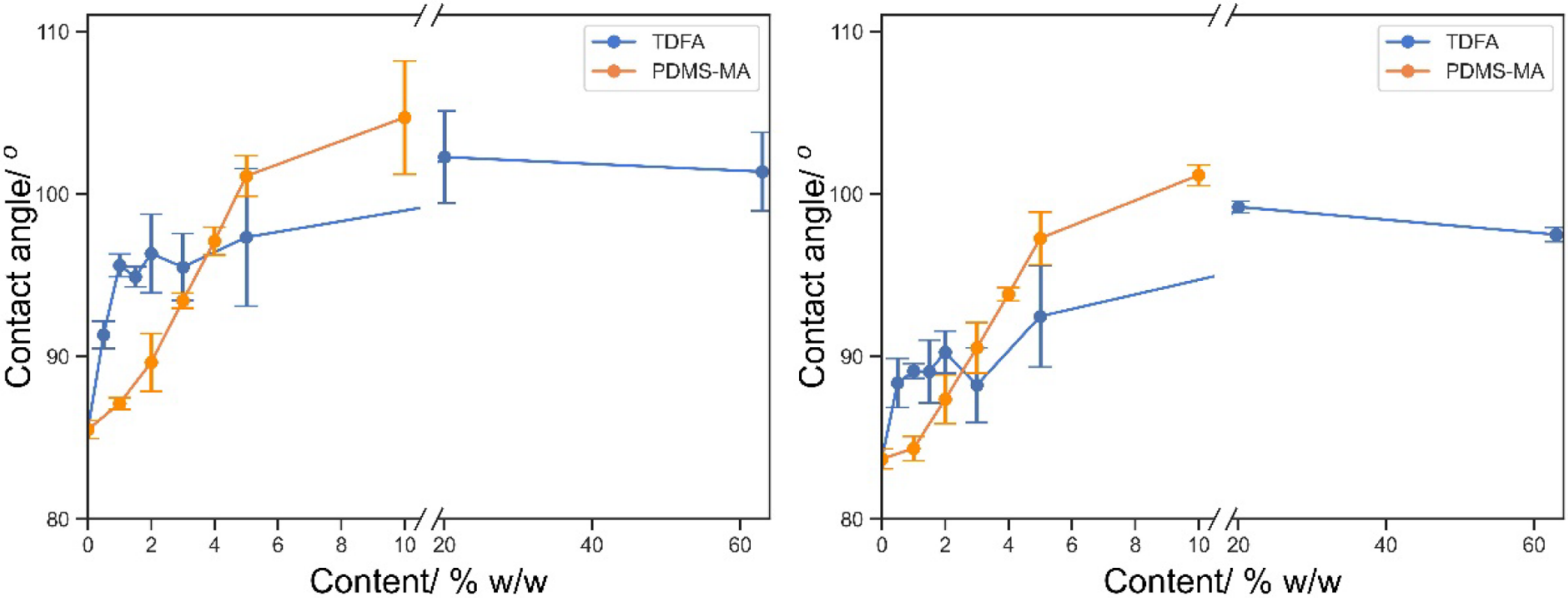
Advancing (left) and receding (right) angles of water on 3D-printed flat surfaces with various contents of TDFA (blue) and PDMS-MA (orange).

To compare the significance of hydrophobicity, isobornyl acrylate was chosen as the base monomer, which is widely used in commercial resins and therefore reflects the necessity of an additional monomer more practically. The contact angle measurements of the 3D-printed flat surfaces are summarized in Tables 3 and 4 and Figure 1. At 0% fluorinated acrylate, the advancing and receding angles were 85° and 83°, respectively. As the concentration of fluorinated acrylate was increased, the contact angles also increased, suggesting that the surface became increasingly hydrophobic. The contact angles reached a plateau at 5% fluorinated acrylate, with values of 100.5° and 99.6° for advancing and receding angles, respectively, with no significant difference even when the content of fluorinated acrylate increased to 20%. The critical concentration and the plateaued contact angles agreed with Swartzfager in copolymers of butyl methacrylate, styrene, butyl acrylate, hydroxypropyl acrylate and (perfluoroalkyl)ethyl methacrylate.^38^ A similar effect was observed when PDMS-MA was used as an auxillary hydrophobic monomer. The contact angle steadily increased from 85° to 107° as 5% w/w PDMS-MA was introduced, then plateaued at even higher concentrations. The range of surface tension of the printed parts was calculated from both advancing and receding angles (numerical approximation for the equilibrium contact angle was not suggested due to θ > 90°). As the content of TDFA increases, γ of the printed parts reduced from 32.1 mN m^–1^ at 0% TDFA to 22.2 mN m^–1^ at 63% TDFA. A similar effect was also observed in PDMS-MA, where γ reduced to 20.2 mN m^–1^ at 10% PDMS-MA.

**Table 3:** Mean and Standard Deviations of Advancing and Receding Angles of Water on 3D- Printed Flat Surface with 0, 0.5, 1, 1.5, 2, 3, 5, 20, 63% w/w of TDFA

TDFA/ % w/w	Advancing angle/ ^o^	Receding angle/ ^o^	γ/ mN m^–1^
0	85.5 (0.56)	83.7 (0.61)	32.1–33.2
0.5	91.3 (0.85)	88.4 (1.50)	28.4–30.2
1	95.6 (0.70)	89.1 (0.46)	25.7–29.8
1.5	94.9 (0.62)	89.1 (1.92)	26.2–29.8
2	96.3 (2.42)	90.3 (1.29)	25.3–29.0
3	95.5 (2.07)	88.2 (2.30)	25.8–30.3
5	97.3 (4.23)	92.5 (3.11)	24.7–27.7
20	102.3 (2.84)	99.2 (0.36)	21.6–23.5
63	101.4 (2.41)	97.5 (0.44)	22.2–24.6

**Table 4:** Mean and Standard Deviations of Advancing and Receding Angles of Water on 3D- Printed Flat Surface with 0, 1, 2, 3, 4, 5, 10% w/w of PDMS-MA

PDMS-MA/ % w/w	Advancing angle/ ^o^	Receding angle/ ^o^	γ/ mN m^–1^
0	85.5 (0.56)	83.7 (0.61)	32.1–33.2
1	87.1 (0.36)	84.3 (0.75)	31.0–32.8
2	89.6 (1.79)	87.4 (1.48)	29.5–30.8
3	93.4 (0.47)	90.5 (1.55)	27.1–28.9
4	97.1 (0.87)	93.8 (0.40)	24.8–26.9
5	101.1 (1.25)	97.3 (1.63)	22.4–24.7
10	104.7 (3.49)	101.2 (0.64)	20.2–22.3

### Exploration of Other Fluorinated Monomers

We also tested other fluorinated monomers to investigate the effect of the side chains and/or monomer type. For comparison, tridecafluorooctyl methacrylate (TDFMA), 2,2,2-trifluoroethyl acrylate (TFEA), and 2,2,2-trifluoroethyl methacrylate (TFEMA) were also incorporated at 5% w/w. The composition and theoretical fluorine content are summarized in Table 5.

**Table 5:** Theoretical F Content in Resin Composed of 5 wt %/w TDFA, TDFMA, TFEA, and TFEMA

Monomer	Mass/ %	Molar % (of monomer)	F content/ % w/w
TDFA	5	2.60	2.95
TDFMA	5	2.52	2.85
TFEA	5	6.75	1.85
TFEMA	5	6.22	1.70

**Figure 2:**
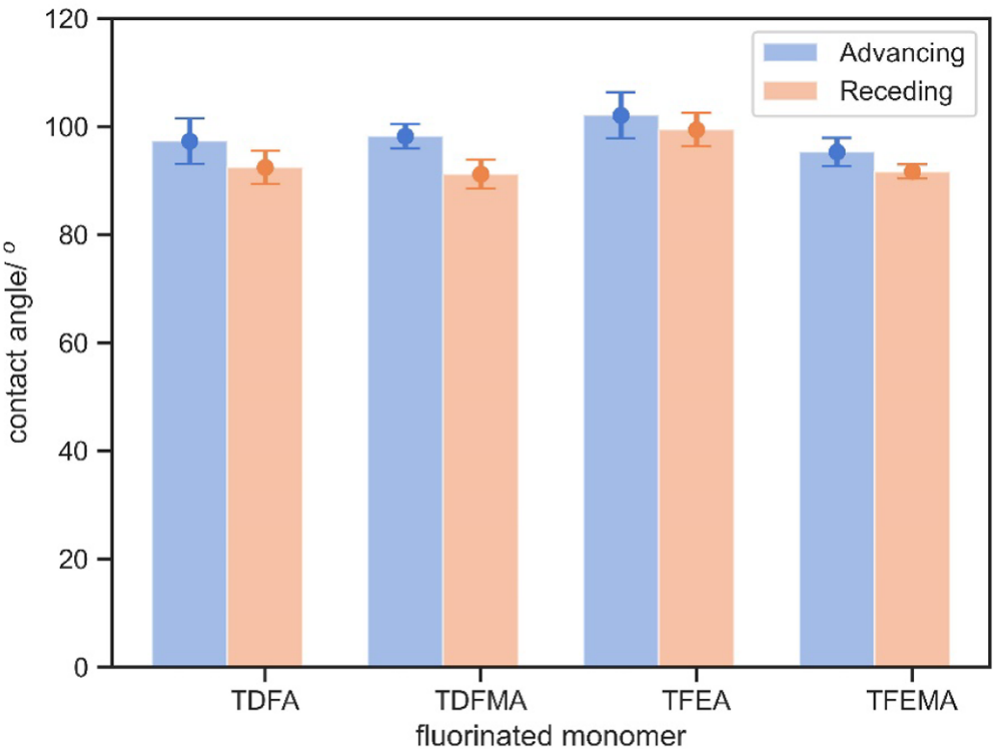
Advancing and receding angles of water on 3D-printed flat surface incorporating 5% w/w TDF(M)A and TFE(M)A.

The advancing and receding contact angles of the 3D-printed parts with 5% (w/w) fluorinated monomers are summarized in Table 6 and Figure 2. We see very little difference between TDF(M)A and TFE(M)A despite the fact that TFE(M)A (CF_3_–CH_2_– side group) contributed a lower F content in total than TDF(M)A (C_6_F_13_–C_2_H_4_- side group). However, it is noted that with the same substituted group, the methacrylate shows a smaller contact angle than its equivalent acrylate, thus, a lower surface tension.

**Table 6:** Mean and Standard Deviations of Advancing and Receding Angles of Water on 3D Printed Flat Surfaces with 5% w/w TDF(M)A and TFE(M)A

Monomer	Advancing ^o^	Receding ^o^	γ/mN m^–1^
TDFA	97.3 (4.23)	92.5 (3.11)	24.7–27.7
TDFMA	98.2 (2.22)	91.2 (2.65)	24.1–28.5
TFEA	102.1 (4.25)	99.4 (3.09)	21.8–23.4
TFEMA	95.3 (2.63)	91.7 (1.27)	25.9–28.2

### Effects of Pore Sizes on Contact Angle

**Figure 3:**
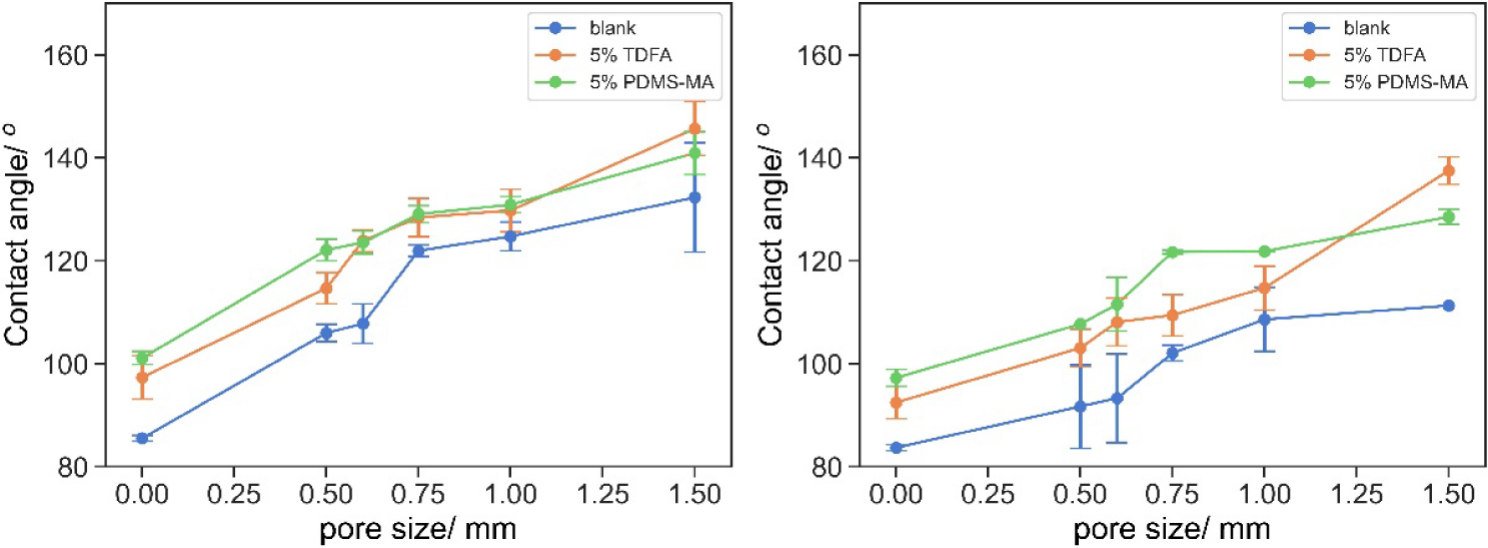
Advancing (left) and receding (right) angles of water on 3D-printed meshes in pore diameters of 0.5, 0.6, 0.75, 1, and 1.5 mm with base resin (blue), 5% w/w TDFA (orange), and 5% w/w PDMS-MA (green).

Surface roughness and porosity are critical factors in considering surface wettability. In this work, we 3D-printed meshes with pores of different diameters with 5% TDFA or PDMS-MA. The spacing between pores was maintained at 0.5 mm longer than the diameter to ensure good printing quality and mechanical robustness. Across all pore sizes, the contact angles of water were greater than those on a flat surface. In particular, the water contact angle of 0.5 mm meshes without any additional hydrophobic monomer was already larger than that for the flat surface composed of 63% w/w TDFA. Considering the effect of pore size, it was found that the contact angle increased with the pore size (Figure 3). This was attributed to the competition of the interfacial tension between the water–air and water–polymer interfaces, and the droplets were pinned to the boundary of the pore, without allowing them to pass through.

### Chemical Composition

**Figure 4:**
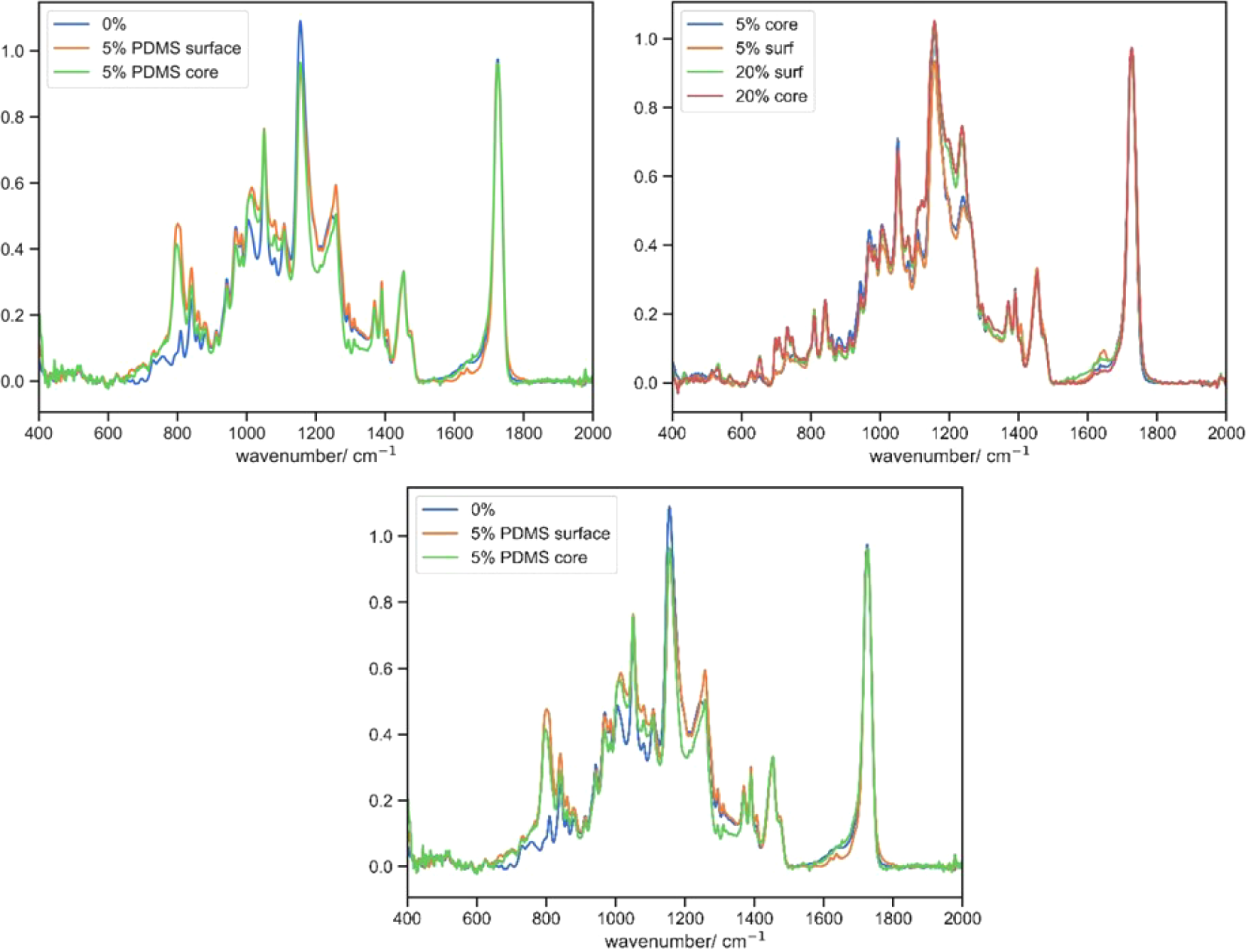
FTIR spectra of 3D-printed parts with the base resin, 5% w/w and 20% w/w TDFA (left), and the same parts measuing from the surface and core (right).

FTIR was used to characterize the chemical composition of the 3D-printed parts (Figure 4 and Table 7). The introduction of TDFA resulted in additional bands at 698 and 731 cm^–1^ and an enhanced intensity at 1237 cm^–1^. We attempted to see if there was any segregation of fluorine at the surface or in the core of the material, exploiting the microscale penetration of FTIR. However, no significant difference was found by acquiring the IR spectra at the surface or beneath the surface. A similar effect was observed in 5% PDMS-MA, where the band of Si–O–CH_3_ rocking appeared sharply, indicating the successful incorporation of the functional monomer. No significant difference was found between the surface and core of the printed material.

**Table 7:** Absorption Bands and their Assignment of FTIR Spectra of the 3D Printed Parts^a^

Wavenumber/cm^–1^	Assignments	References
2952	Asym C–H stretch (CH_3_)	(39)
2876	Sym C–H stretch (CH_3_)	(40)
1726	C=O stretching	(40)
1454	C–H def.	(39)
1390	C–(CH_3_) sym def.	(41)
1371	C–(CH_3_) sym def.	(40)
1237	–C–C–O stretching	(42)
1237*	C–F stretching	(43)
1157	C–O–C stretching	(41)
1050	Sym C–O–C stretch	(41)
798	Si–O–CH_3_ rocking	(44)
731	CF_2_ rocking	(45)
698	CF_2_ wagging	(45)

a *: The wavenumber suggested in ref. (6) was 1210 cm^–1^ but it overlaps and enhanced the band at 1237 cm^–1^.

### Atom Distribution by SEM-EDX

**Figure 5:**
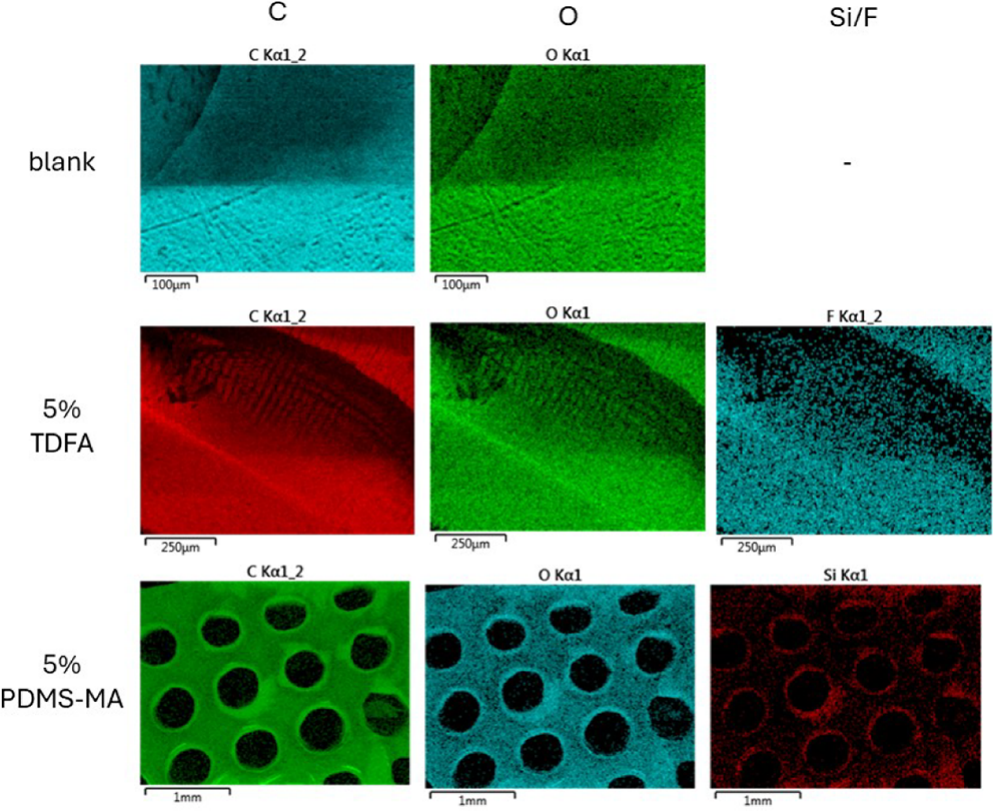
Color mapping of C, O, F/Si distribution of the 3D-printed parts with the base resin, 5% w/w TDFA, and 5% w/w PDMS-MA by SEM-EDX.

SEM-EDX was used to determine the elemental composition and distribution in the 3D-printed parts (Figure 5). The composition by XPS is summarized in Figure 5, with the F content among C/O/F (*note that H is not observed by EDX*) at 3.3% w/w, which agreed with the theoretical value of 3.22. However, this contrasted to the findings by Swartzfager et al., who reported the enrichment of fluorine at the air interface by 3 times the inner depth in thin films of poly(butyl methacrylate), poly(butyl acrylate), poly(hydroxypropyl methacrylate), and polystyrene with up to 6% w/w (perfluoroalkyl)ethyl methacrylate.^38^ This was further supported by EDX mapping, with a homogeneous distribution of F atoms observed in the cross-section of the 3D-printed parts. A similar result was observed in the case of PDMS-MA, with Si atoms distributed homogeneously without any surface enrichment observed. We postulated that although fluorinated carbons and PDMS have a low air surface tension and tend to migrate to the object surface, the polymers in 3D printing are highly cross-linked; therefore, the mobility to segregate and enrich at the surface was severely restricted, as opposed to the solution-cast film of linear polymer in the Swartzfager study.

### Oil–Water Separation by 3D-Printed Mesh

**Figure 6:**
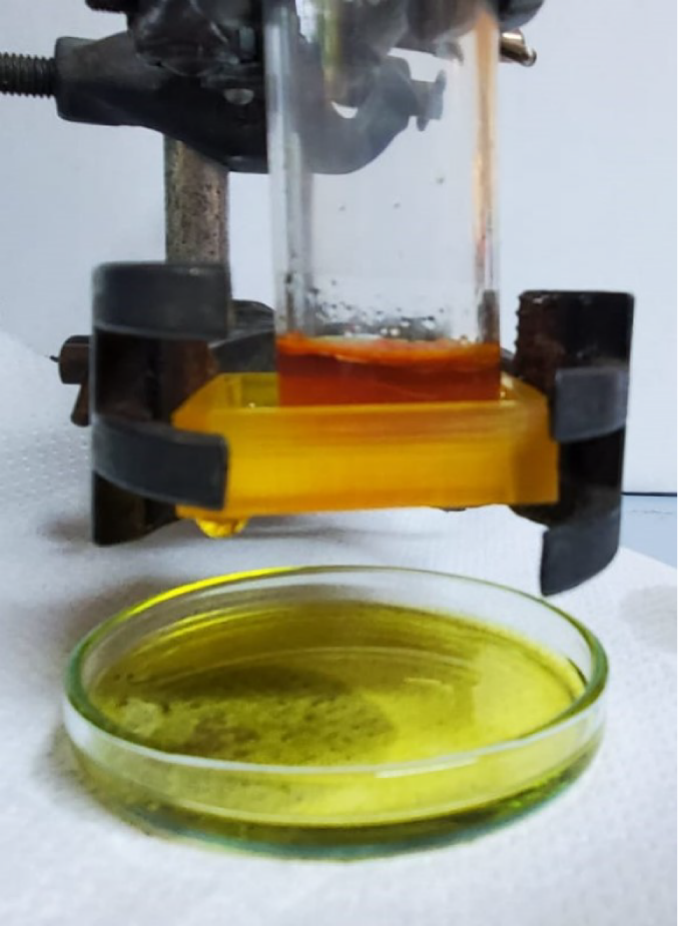
Typical image of successful oil–water separation by 3D-printed mesh; Alizerin-dyed water was retained by the mesh, whereas oil passed through. Photograph courtesy of “Wai Hin Lee”, Copyright 2024.

3D-printed meshes with pore sizes of 0.5, 0.6, 0.75, 1, and 1.5 mm were printed with the base resin, 5% TDFA, or 5% PDMS-MA. Alizarin solutions in tributyrin and water were prepared, giving a yellow appearance in the organic phase and pink in the aqueous phase after the ring opening of the sulfonate. It was observed that only the yellow tributyrin phase passed through the mesh, while the pink aqueous phase was retained (Figure 6).

We would have anticipated that a larger pore size is preferable to allow a faster flow rate with a reduction in the back pressure if additional force is applied. To evaluate the separation efficiency, tributyrin and water were passed through the 3D-printed meshes with increasing pore sizes until the separation failed. Then, the fraction of tributyrin and water was measured (Table 8).

**Table 8:** Separation Efficiency of 3D-Printed Meshes with Various Compositions and Pore Sizes

	blank		5% TDFA		5% PDMS-MA	
Pore size	*m*_*oil*_	*m*_*water*_	*m*_*oil*_	*m*_*water*_	*m*_*oil*_	*m*_*water*_
0.5	40.7%		63.4%		93.2%	
0.6	>99%		>99%		91.8%	
0.75	>99%	8.88%	>99%	15.3%	>99%	
1	Failed to separate oil and water					
1.5						

The base resin and 5% TDFA showed similar results. At a 0.5 mm pore size, tributyrin only partially passed through the mesh, whereas complete passage was allowed at larger pore sizes. However, the aqueous phase started to pass through at a 0.75 mm pore size (8.88% and 15.3% for blank and 5% TDFA, respectively), and it failed to separate at 1 and 1.5 mm pore sizes. The separation efficiency improved significantly with 5% PDMS-MA, where complete passage was attained even at a 5 mm pore size, and the water phase remained completely separated at 0.75 mm. Complementary to the contact angle measurement, this demonstrated that the surface geometry played a crucial role in the separation efficiency, whereas the chemical composition was less significant, despite the enhancement of hydrophobicity by PDMS or the fluorinated monomer, when the base resin (isobornyl acrylate in this case) already offers certain hydrophobicity.

## Conclusions

We report the use of DLP 3D printing using inexpensive consumer printers to incorporate hydrophobic silicone- and fluorine-containing monomers into polymer materials and compare chemical modification with these monomers and surface geometry engineering. For chemical modification, it was found that the contact angle increased with the content of both fluorinated and PDMS monomers up to a maximum of 5% for both types of monomers. The effect of surface geometry was also investigated such that the contact angle increased with the pore size. Lastly, to evaluate the significance of each approach in practice, we 3D printed meshes with different pore sizes with base resin, fluorinated monomer, or PDMS monomer and used them to perform oil–water separation. It was found that PDMS-modified polymers gave the best separation efficiency; however, no significant improvement was seen with fluorinated monomers. This highlights the importance of design in surface geometry, which can be beneficial over the use of environmentally unfriendly and potentially toxic fluorinated- or silicone-based monomers.

## Supporting Information Available

Contact angle images (PDF)
