# Supplementary material for: Digital Light Processing (DLP) 3D Printing Fabrication of Hydrophobic Meshes Incorporating Fluorinated and Silicone-Based Acrylates Combined with Surface Engineering: Comparison of Their Oil–Water Separation Efficiency
Source: ACS Omega. 2024 Nov 29;9(50):49463–9. doi: 10.1021/acsomega.4c07193 (PMC11656361; doi:10.1021/acsomega.4c07193)
Supplement: Supplementary file 1 — ao4c07193_si_001.pdf [file ao4c07193_si_001.pdf]

Digital light processing (DLP) 3D printing fabrication of hydrophobic meshes incorporating fluorinated and silicone-based acrylates combined with surface engineering: comparison of their oil-water separation efficiency.

Wai Hin, Lee and David, Haddleton**

Department of Chemistry, University of Warwick, Coventry, CV4 7AL

Email: D.M.Haddleton@warwick.ac.uk, wai-hin.lee.1@warwick.ac.uk

Supplementary information

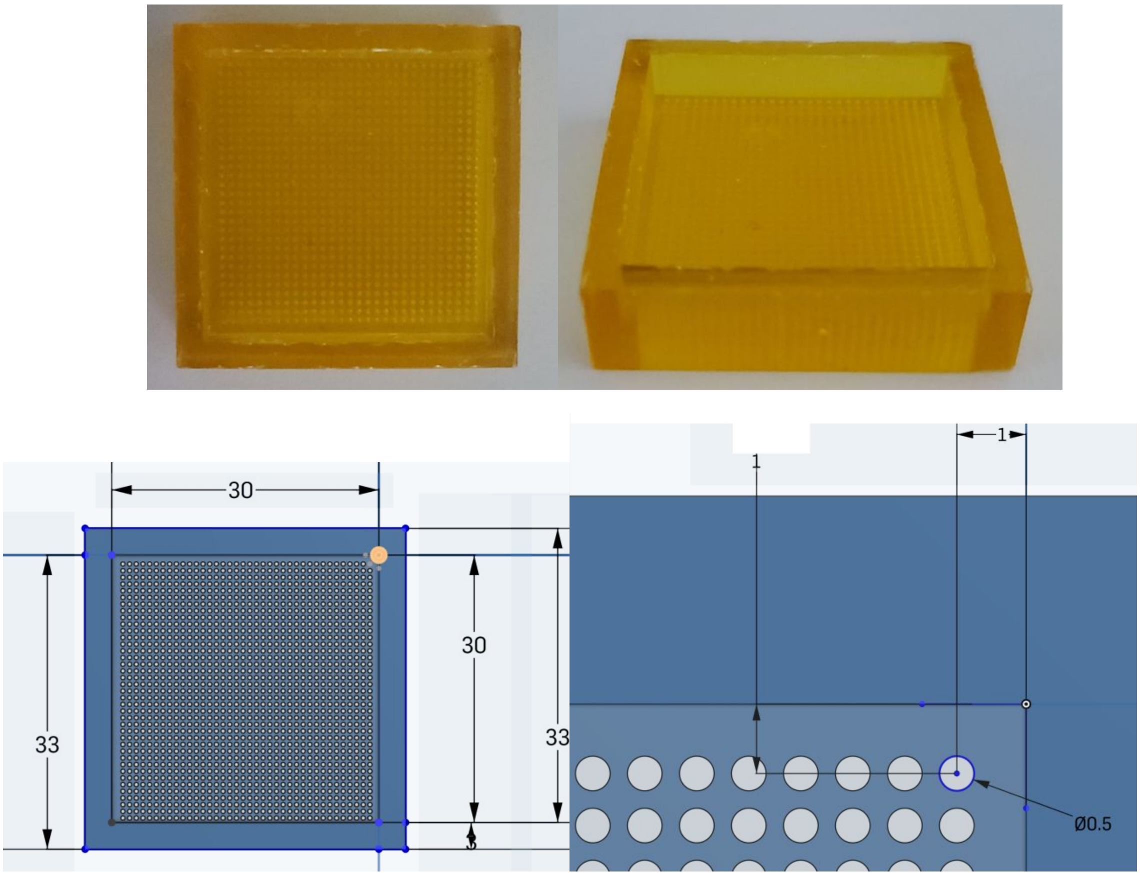

Figure S1 Typical photo of the 3D printed mesh (pore diameter= 0.5 mm) and the stl.file with the indicated length (in mm)

	Advancing angle	Receding angle
0%	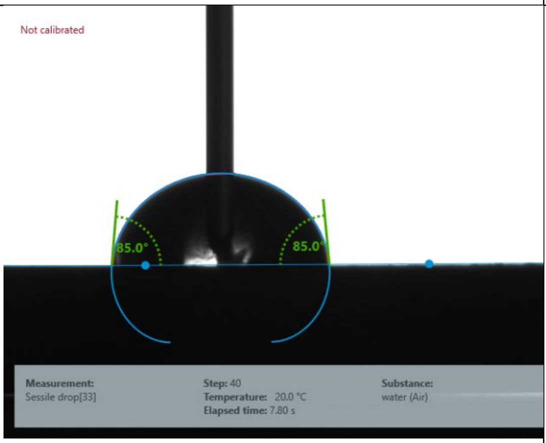	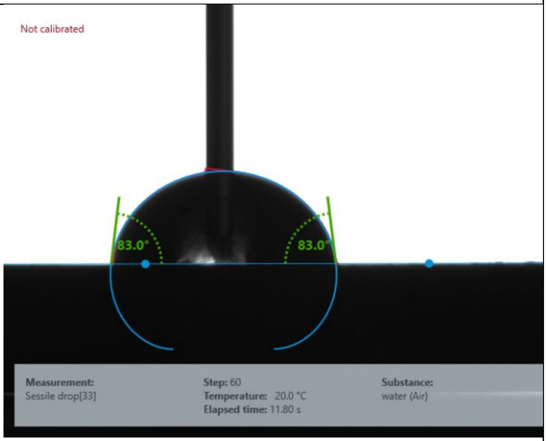
0.5%	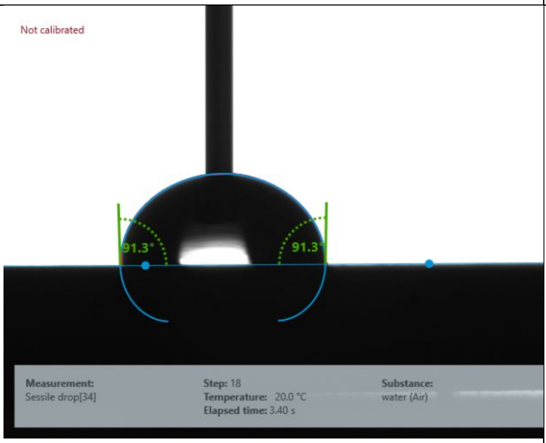	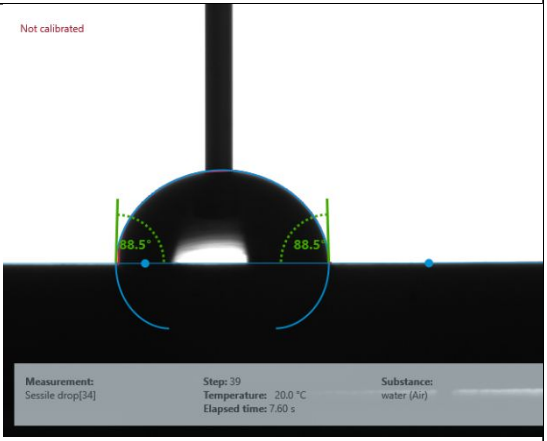
1%	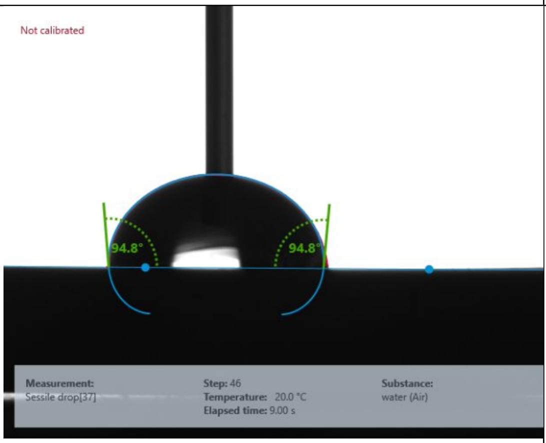	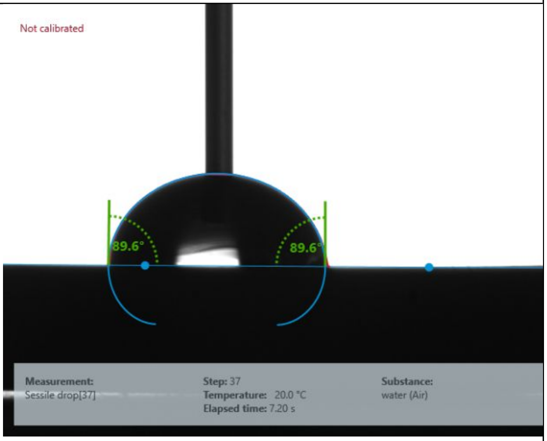
1.5%	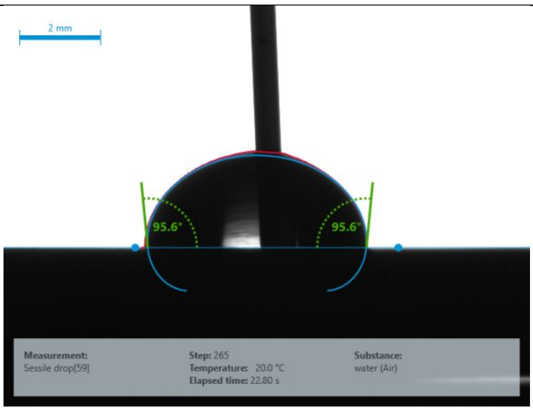	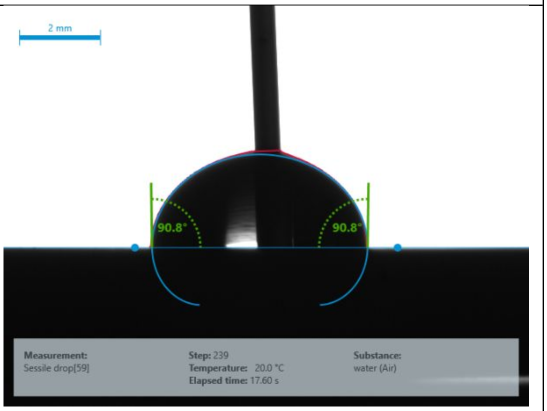

2%

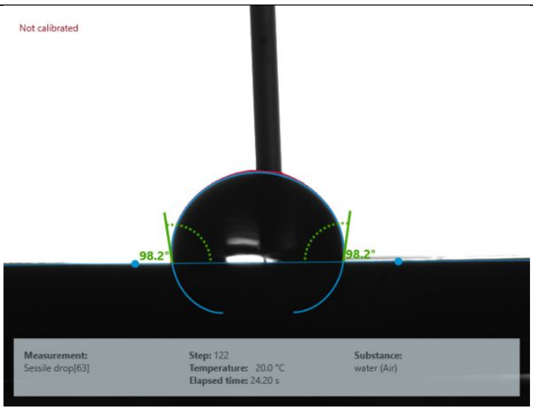
Not calibrated
98.2°
98.2°
Measurement:
Sessile drop[63]
Step: 122
Temperature: 20.0 °C
Elapsed time: 24.20 s
Substance:
water (Air)

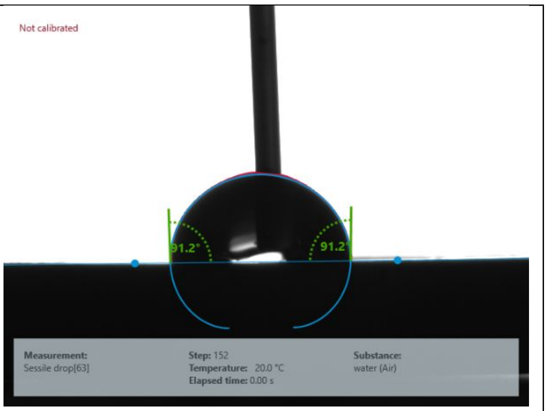
Not calibrated
91.2°
91.2°
Measurement:
Sessile drop[63]
Step: 152
Temperature: 20.0 °C
Elapsed time: 0.00 s
Substance:
water (Air)

3%

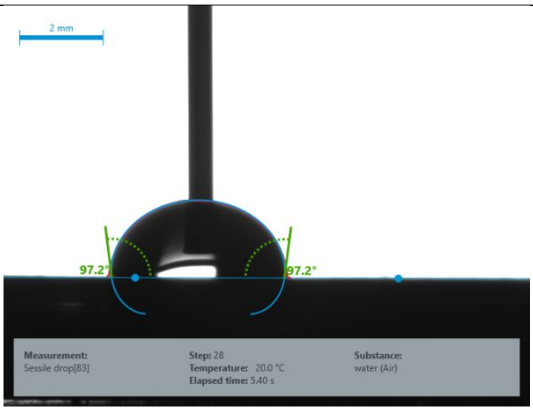
2 mm
97.2°
97.2°
Measurement:
Sessile drop[83]
Step: 28
Temperature: 20.0 °C
Elapsed time: 5.40 s
Substance:
water (Air)

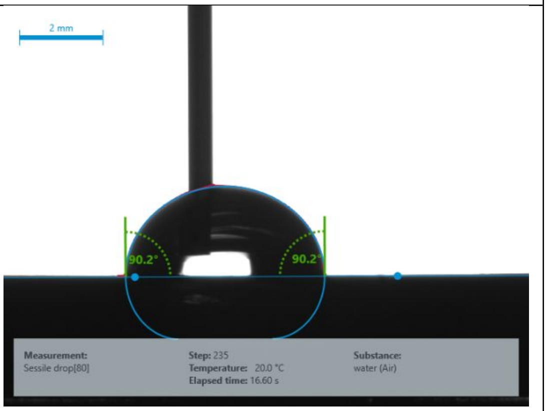
2 mm
90.2°
90.2°
Measurement:
Sessile drop[80]
Step: 235
Temperature: 20.0 °C
Elapsed time: 16.60 s
Substance:
water (Air)

5%

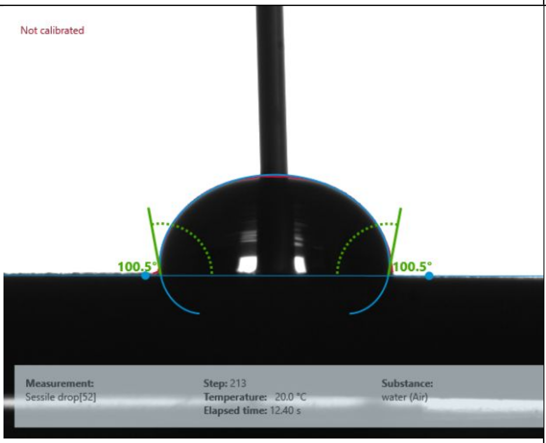
Not calibrated
100.5°
100.5°
Measurement:
Sessile drop[52]
Step: 213
Temperature: 20.0 °C
Elapsed time: 12.40 s
Substance:
water (Air)

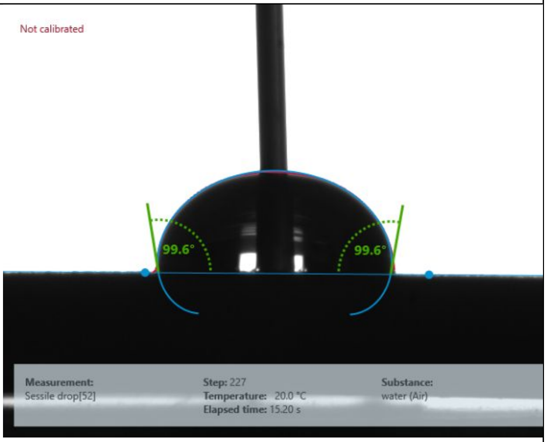
Not calibrated
99.6°
99.6°
Measurement:
Sessile drop[52]
Step: 227
Temperature: 20.0 °C
Elapsed time: 15.20 s
Substance:
water (Air)

20%

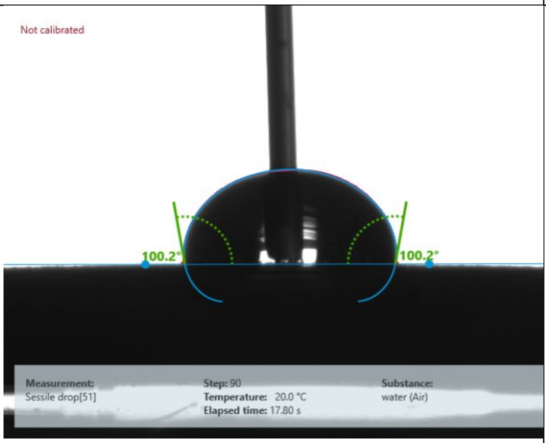
Not calibrated
100.2°
100.2°
Measurement:
Sessile drop[51]
Step: 90
Temperature: 20.0 °C
Elapsed time: 17.80 s
Substance:
water (Air)

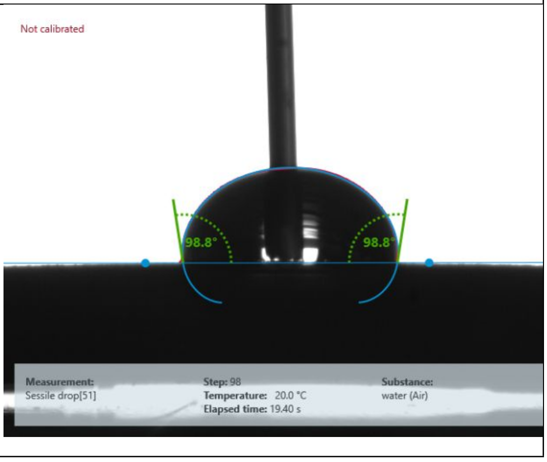
Not calibrated
98.8°
98.8°
Measurement:
Sessile drop[51]
Step: 98
Temperature: 20.0 °C
Elapsed time: 19.40 s
Substance:
water (Air)

63%	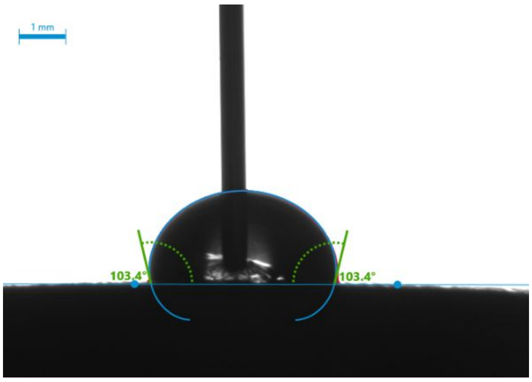	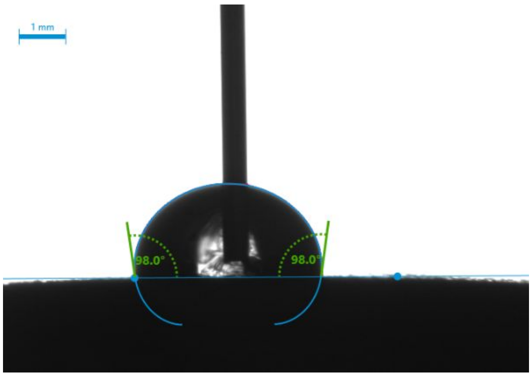

Figure S2 Advancing and receding angles of water on 3D printed flat surface with 0, 0.5,1,2,3,5,10,63% w/w TDFA

<table>
<tr><th></th><th>Advancing º</th><th>Receding º</th></tr>
<tr><td>1%</td><td>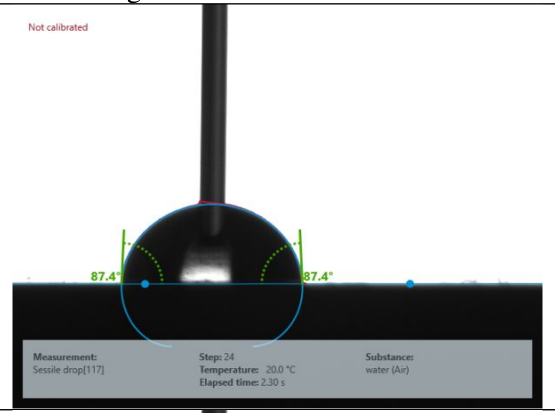
</td><td>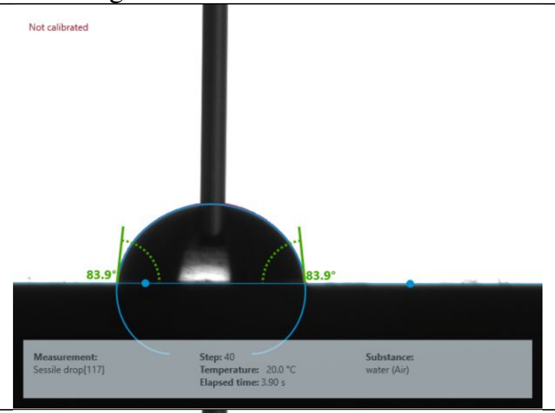
</td></tr>
<tr><td>2%</td><td>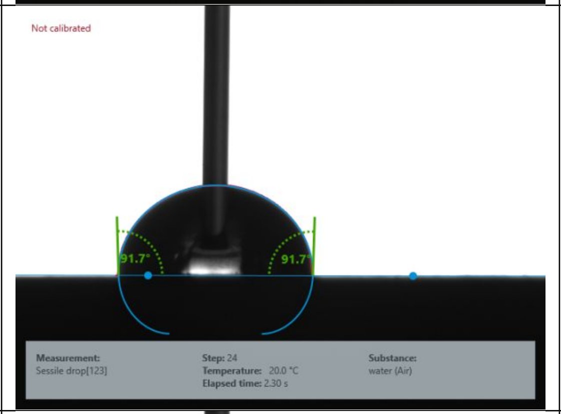
</td><td>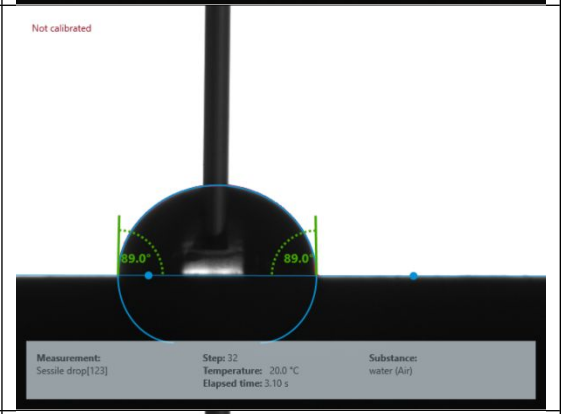
</td></tr>
<tr><td>3%</td><td>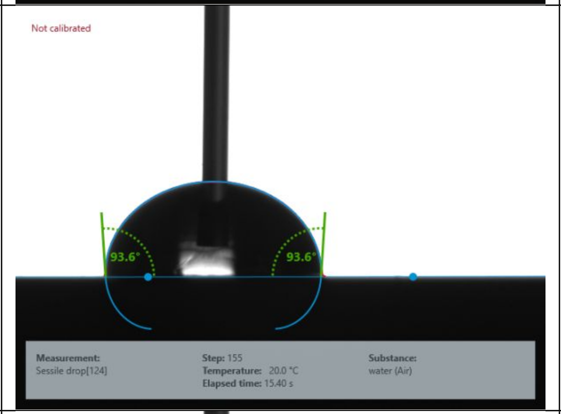
</td><td>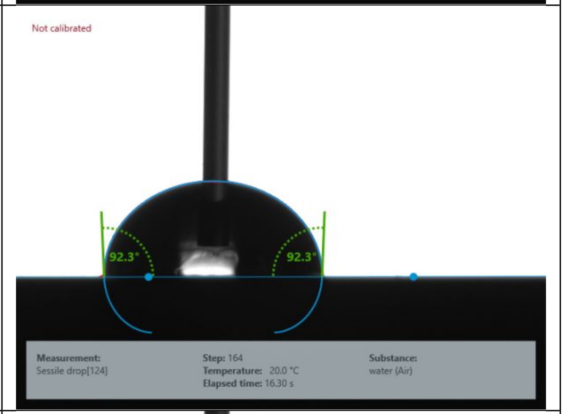
</td></tr>
<tr><td>4%</td><td>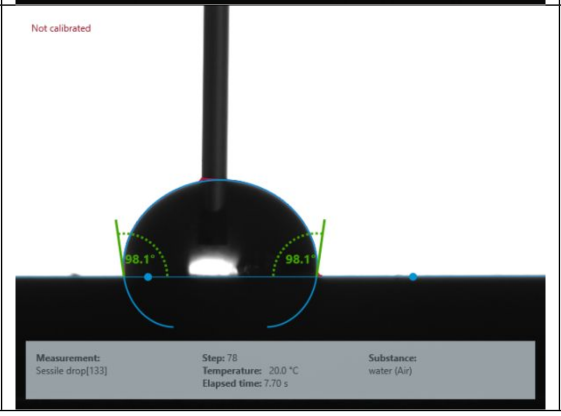
</td><td>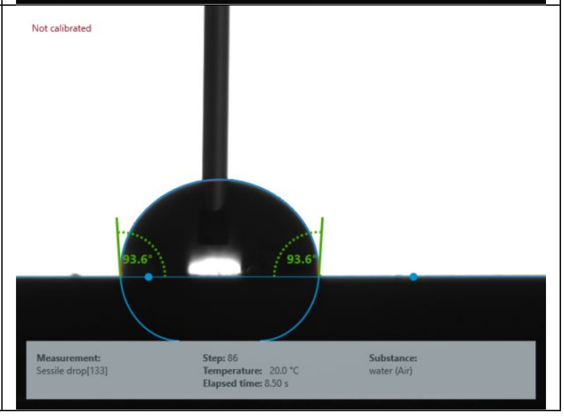
</td></tr>
</table>

5%

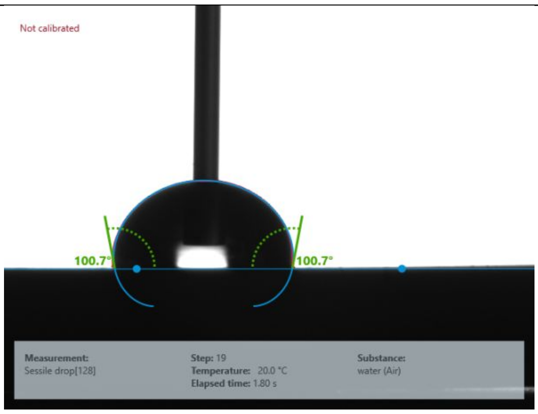

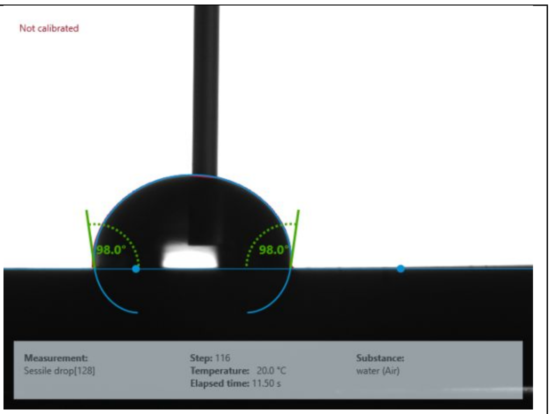

10%

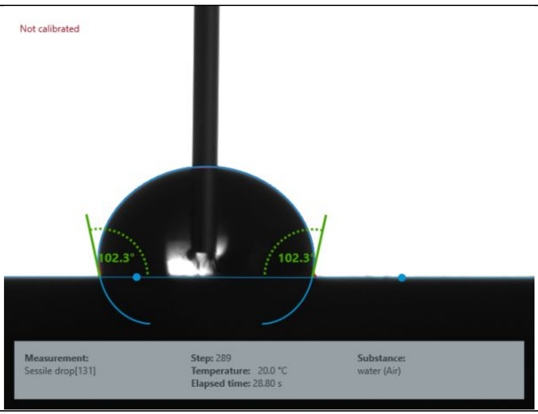

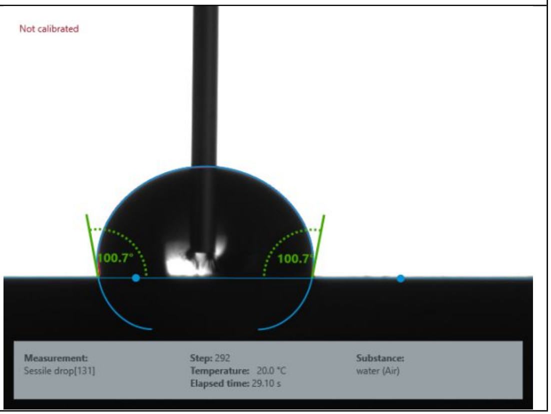

Figure 1S3 Advancing and receding angles of water on 3D printed flat surface with 1, 2, 3, 4, 5, 10% w/w PDMS-MA

Monomer	Advancing °	Receding °
TDFA	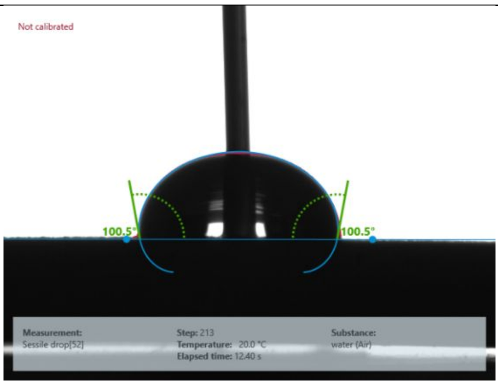	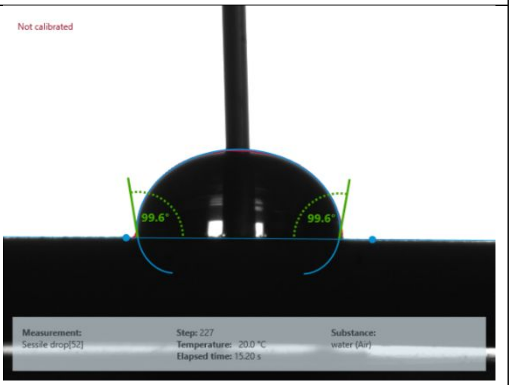
TDFMA	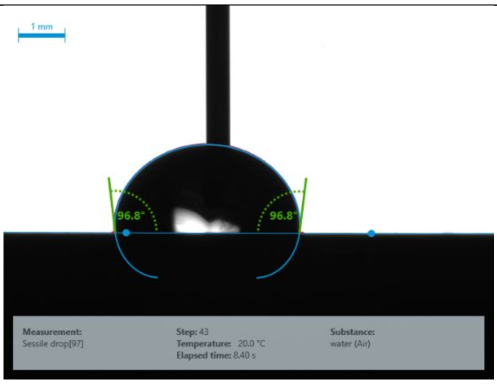	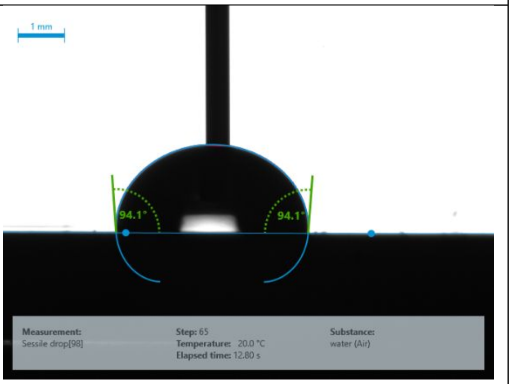
TFEA	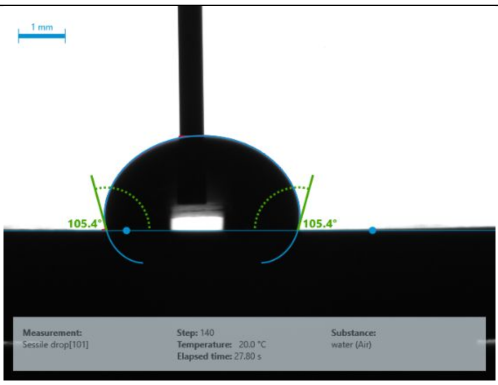	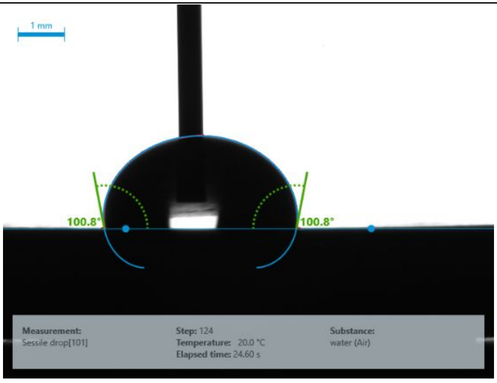
TFEMA	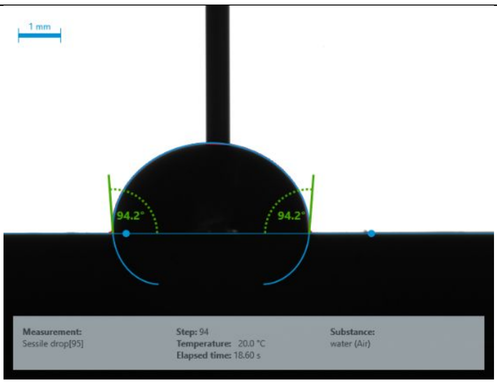	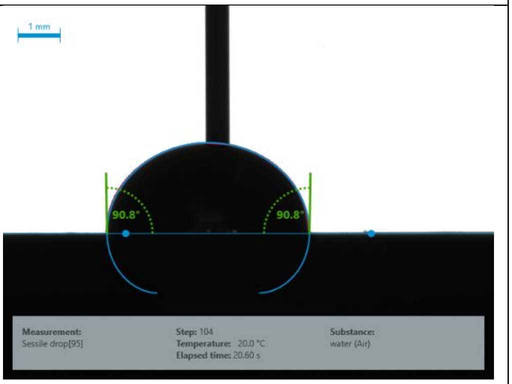

Figure S4 Advancing and receding angles of water on 3D printed flat surface 5% w/w TDFA,TDFMA,TFEA,TFEMA

Pore size /mm	Advancing °	Receding °
0.5	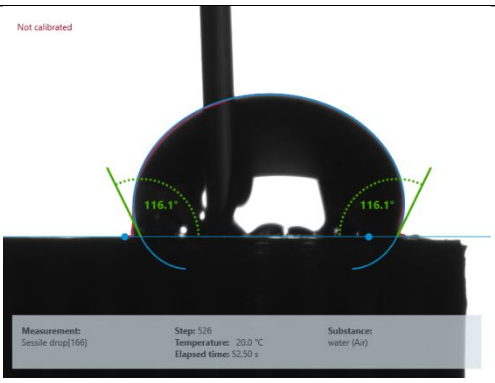	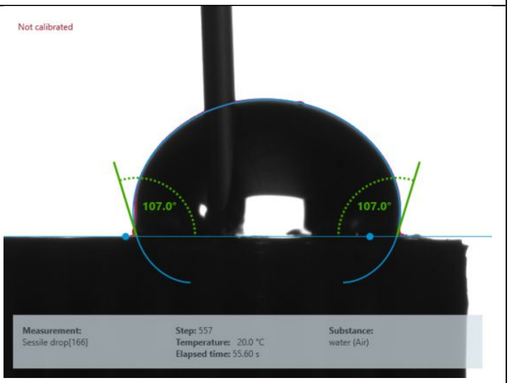
0.6	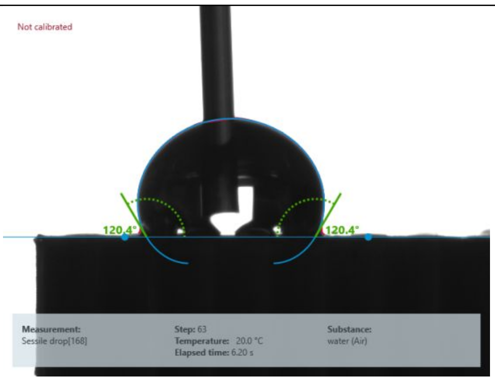	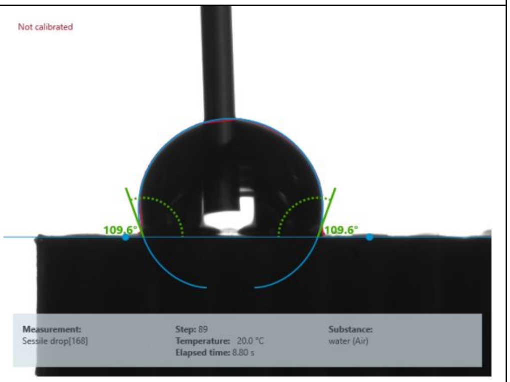
0.75	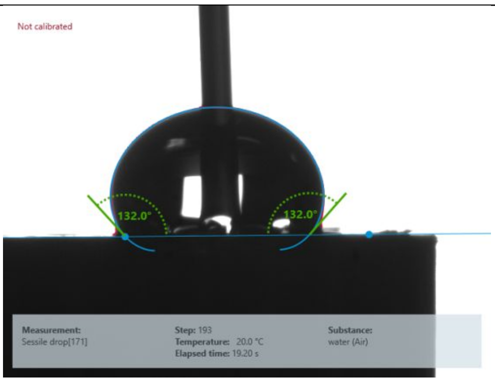	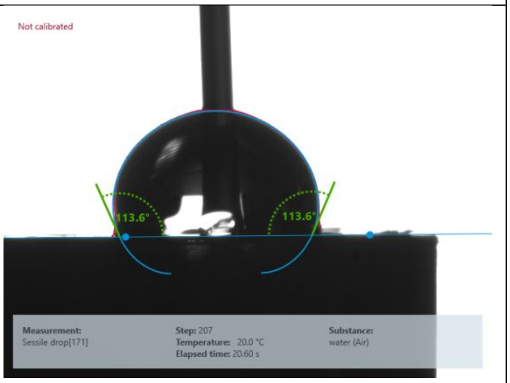
1.0	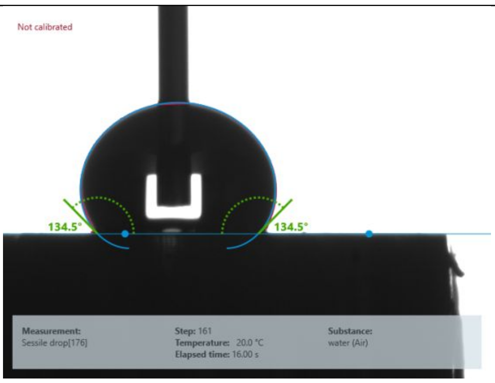	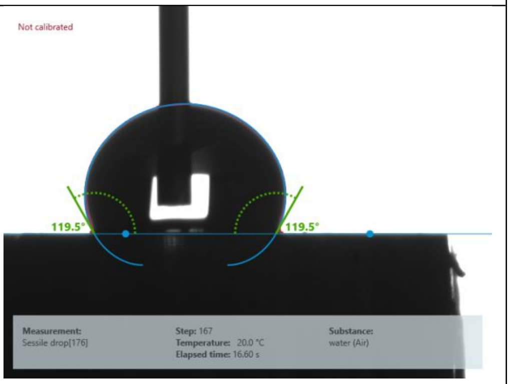

1.5	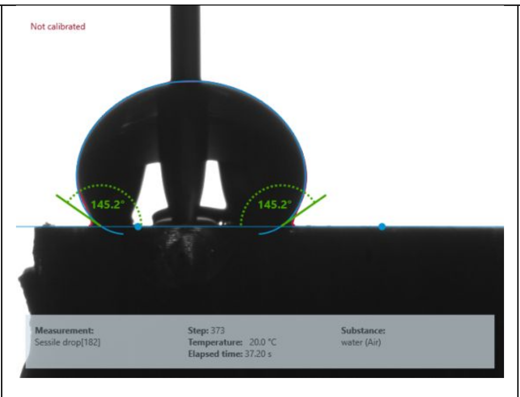	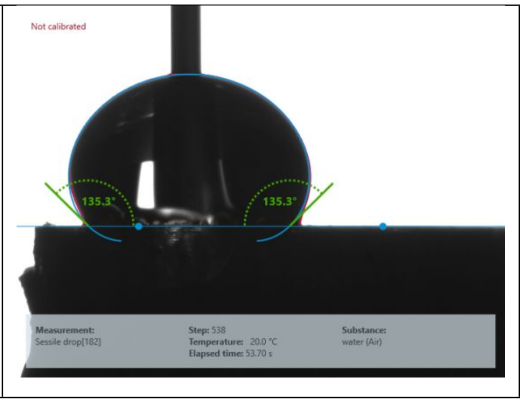

Figure S52 Advancing and receding angles of water on 3D printed meshes composed of 5% w/w TDFA with the pore diameter of 0.5, 0.6, 0.75, 1, 1.5 mm respectively

Pore size /mm	Advancing °	Receding °
0.5	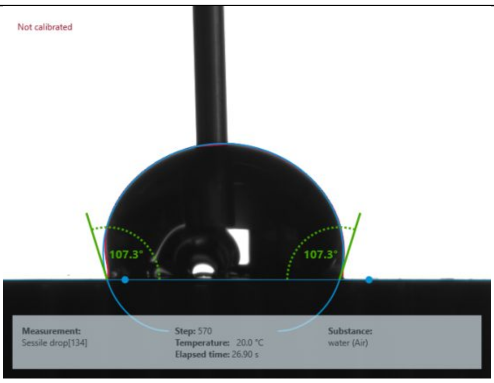	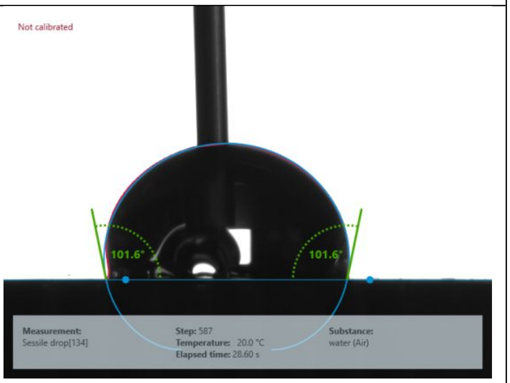
0.6	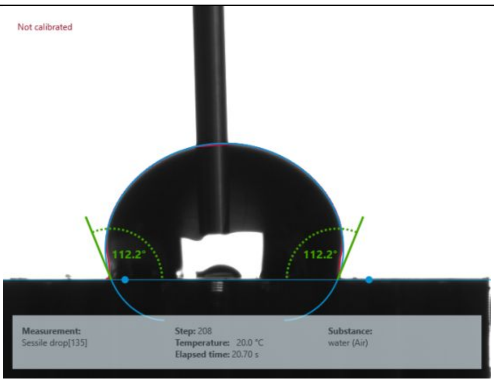	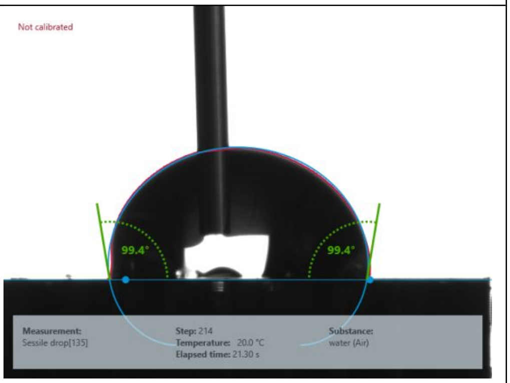
0.75	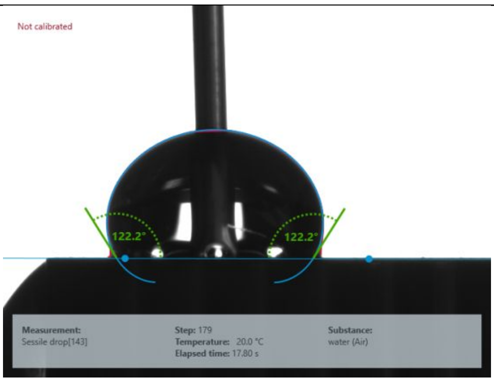	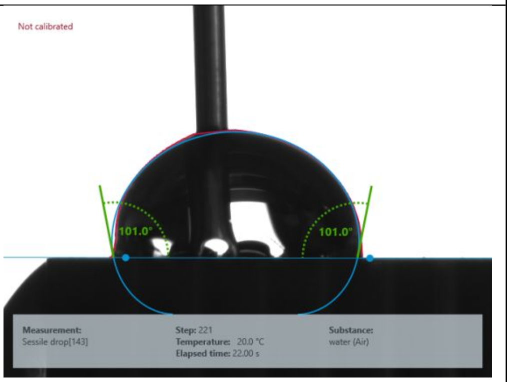
1.0	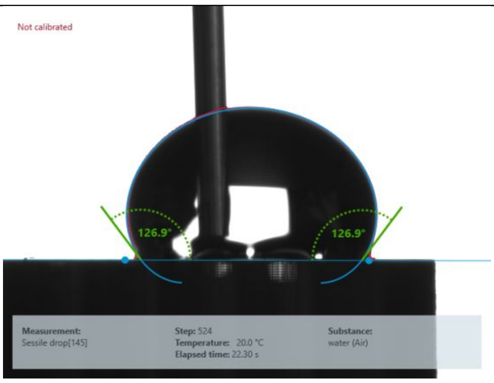	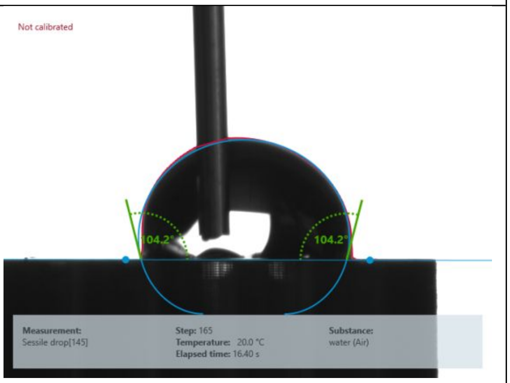

1.5	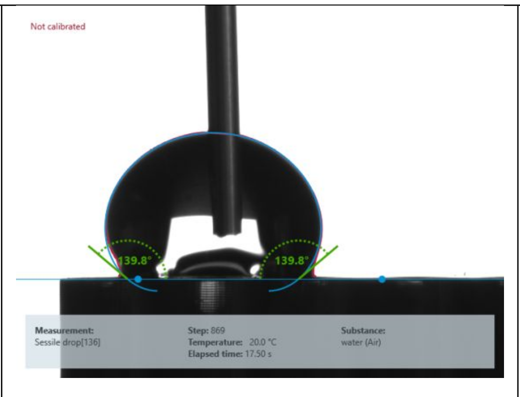	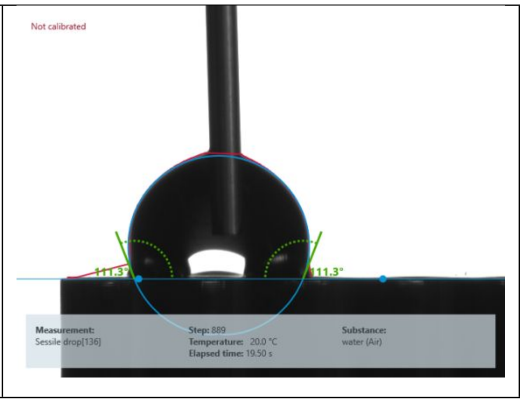

Figure S6 Advancing and receding angles of water on 3D printed meshes composed of base resin with the pore diameter of 0.5, 0.6, 0.75, 1, 1.5 mm respectively

<table>
<tr><th>Pore size
/mm</th><th>Advancing °</th><th>Receding °</th></tr>
<tr><td>0.5</td><td>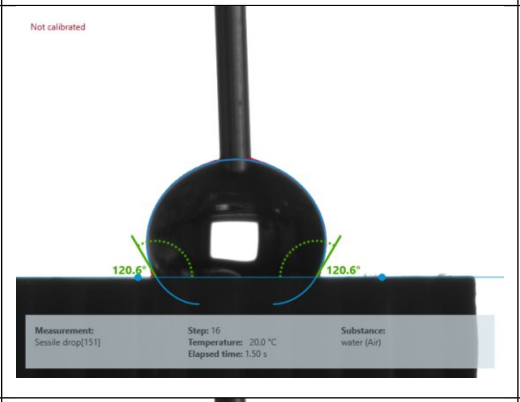
</td><td>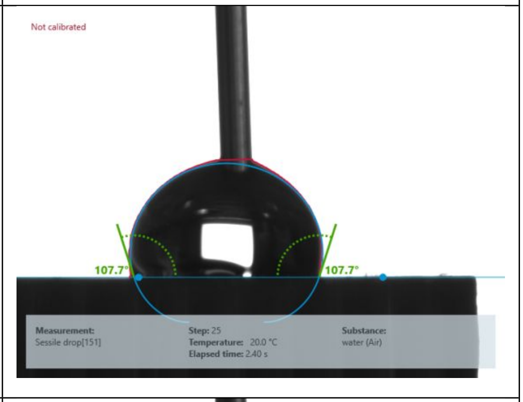
</td></tr>
<tr><td>0.6</td><td>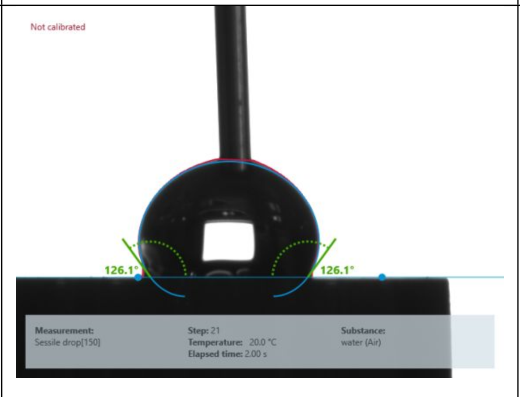
</td><td>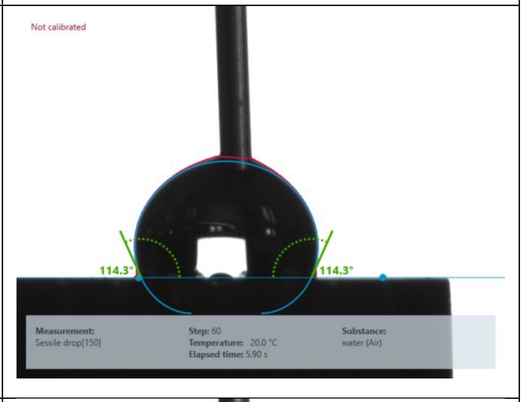
</td></tr>
<tr><td>0.75</td><td>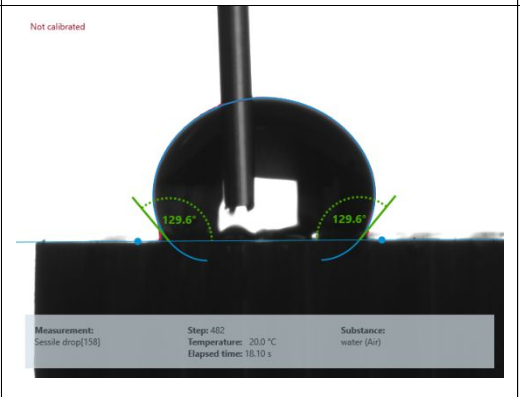
</td><td>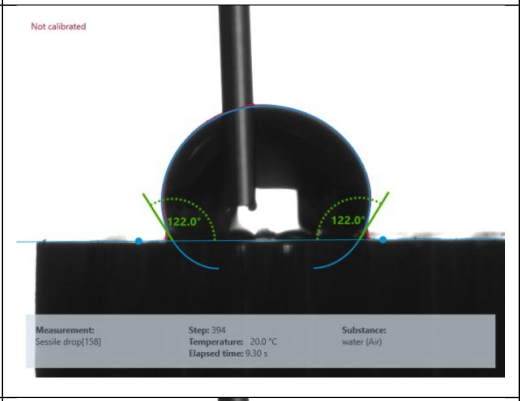
</td></tr>
<tr><td>1.0</td><td>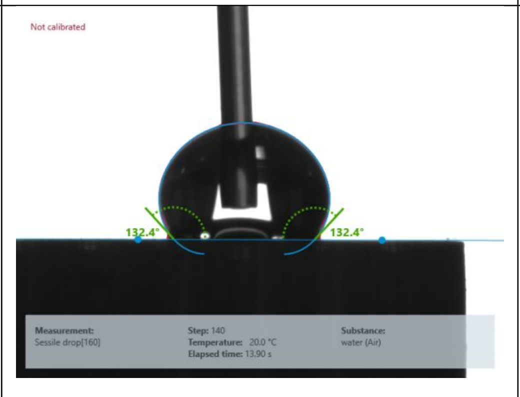
</td><td>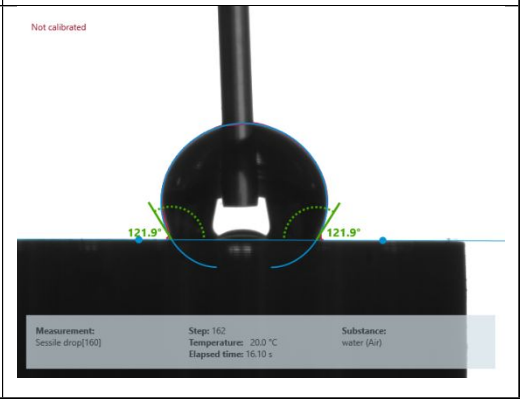
</td></tr>
</table>

<table>
<tr>
<td>1.5</td>
<td>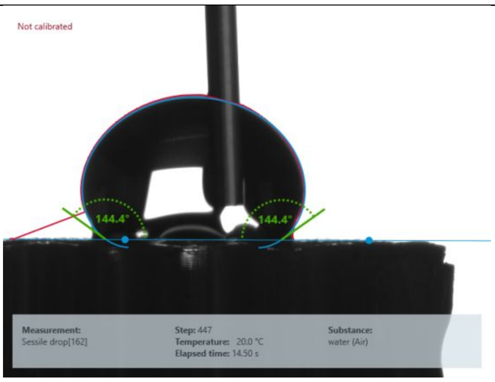
</td>
<td>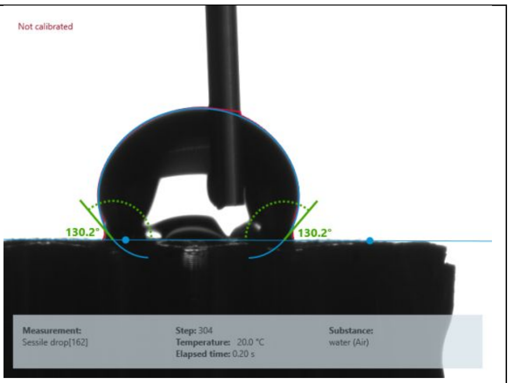
</td>
</tr>
</table>

Figure S7 Advancing and receding angles of water on 3D printed meshes composed of 5% w/w PDMS-MA with the pore diameter of 0.5, 0.6, 0.75, 1, 1.5 mm respectively
